# Passive smoking and risk of gestational diabetes mellitus: A systematic review and meta-analysis

**DOI:** 10.18332/tid/169722

**Published:** 2023-09-15

**Authors:** Haijie Zhang, Xin Zhou, Lixia Tian, Jianjun Huang, Meng E, Jinzhu Yin

**Affiliations:** 1 Shanxi Health Commission Key Laboratory of Nervous System Disease Prevention and Treatment, Sinopharm Tongmei General Hospital, Datong, China; 2 Department of Clinical Nutrition, Sinopharm Tongmei General Hospital, Datong, China; 3 The Innovation Center of Coal Mine Public Health Graduate Student of Shanxi Province Sinopharm Tongmei General Hospital, Datong, China; 4 Yangzhou Center for Disease Control and Prevention, Yangzhou, China; 5 Department of Neurosurgery, Sinopharm Tongmei General Hospital, Datong, China; 6 Department of Central Laboratory, Sinopharm Tongmei General Hospital, Datong, China

**Keywords:** passive smoking, gestational diabetes mellitus, GDM, metaanalysis

## Abstract

**INTRODUCTION:**

Pregestational smoking increases the risk of gestational diabetes mellitus (GDM) and is a common health problem during pregnancy, with its incidence on the rise worldwide, especially in China. This study is a meta-analysis of passive smoking as a risk factor associated with GDM.

**METHODS:**

Two independent reviewers searched passive smoking and the risk of GDM in PubMed, Medline, Web of Knowledge, Science Direct, China National Knowledge Internet (CNKI) and Wanfang databases (up to May 2023). The authors extracted the study data independently and used the Newcastle–Ottawa scale (NOS) to evaluate the quality of the included articles. A meta-analysis was conducted using a random effects model depending on the size of the heterogeneity. Begg’s and Egger’s tests were performed to assess publication bias.

**RESULTS:**

The overall relative risk for GDM caused by passive smoking was 1.47 (95% CI: 1.31–1.64), with moderate heterogeneity between studies (I^2^=41.7%, p=0.079). Subgroup and sensitivity analyses were stable, and no evidence of publication bias was found.

**CONCLUSIONS:**

Passive smoking is a risk factor for GDM, even in those who are not active smokers. To eliminate the effects of other confounding factors, larger prospective cohort studies are required to clarify the relationship between passive smoking and the occurrence of GDM.

## INTRODUCTION

Gestational diabetes mellitus (GDM), which refers to abnormal glucose metabolism first detected or occurring during pregnancy, is a prevalent complication^1^. Survey data show that more than 90% of diabetes in pregnant women is GDM^2^, which is increasing worldwide^3^. GDM has both short- and long-term health effects during pregnancy and subsequent generations. These women are at increased risk of type 2 diabetes^4^, and their offspring are at increased risk of childhood obesity^5^ and adult cardiovascular disease^6^. A meta-analysis has shown that active smoking during pregnancy is associated with an increased risk of GDM^7^ (OR=2.322; 95% CI: 1.359–3.967). However, many pregnant women choose to quit smoking during pregnancy, but passive smoking during pregnancy is also harmful. Studies have shown that passive smoking can increase the risk of type 2 diabetes^8^. However, there is insufficient research to confirm that passive smoking and GDM are associated. This study aims to clarify whether passive smoking is a risk factor for GDM through a systematic review and meta-analysis.

## METHODS

### Search strategy and selection criteria

This meta-analysis was performed according to the Preferred Reporting Item for Systematic Reviews and Meta-analysis (PRISMA) guidelines^9^ (Supplementary file). Published articles were searched on passive smoking and GDM (up to May 2023). English articles were mainly searched in PubMed, Medline, Web of Knowledge, and Science Direct. Chinese articles were searched in the CNKI and Wanfang databases. The search terms were: ‘passive smoking’, ‘secondhand smoking’, ‘environmental smoking’, and ‘gestational diabetes mellitus or GDM’. To avoid omissions, the researchers reviewed references that met the study criteria.

Study selection and extraction criteria: 1) cohort study or case-control study; 2) diagnosis of gestational diabetes or GDM; 3) exposure to passive smoking; and 4) effect size (OR and relative risk, RR), CI, and any information that can be derived.

Exclusion criteria: 1) exposure factors were not identified as passive smoking in the study; 2) reviews, case reports, meetings, letters, and animal studies; and 3) studies without OR values or where the OR and 95% CI could not be calculated in the raw data provided.

### Data extraction and quality assessment

Two researchers extracted authors (year of publication), study type, country, sample size, and number of GDM cases. The OR (RR, HR) and 95% CI were extracted to conduct a meta-analysis and adjust confounding factors. The selected articles were then assessed for quality using the NOS^10^. There are nine entries on this self-rating scale, each occupying 1 point. The quality of the article was independently assessed by HZ and EM based on previous studies; only those with NOS scores ≥5 were selected^10^.

### Statistical analysis

Statistical analysis was performed using Stata 13.0. Judging heterogeneity by I^2^, a low heterogeneity was considered when I^2^ <25.0%. A fixed effects model analysis was used; otherwise, a random effects model was used to calculate the pooled OR^11^. Sources of heterogeneity between studies were explored by sensitivity analysis. Begg’s or Egger’s method and the funnel plot^12^ were used to test for publication bias.

## RESULTS

### Study selection

**Figure 1:**
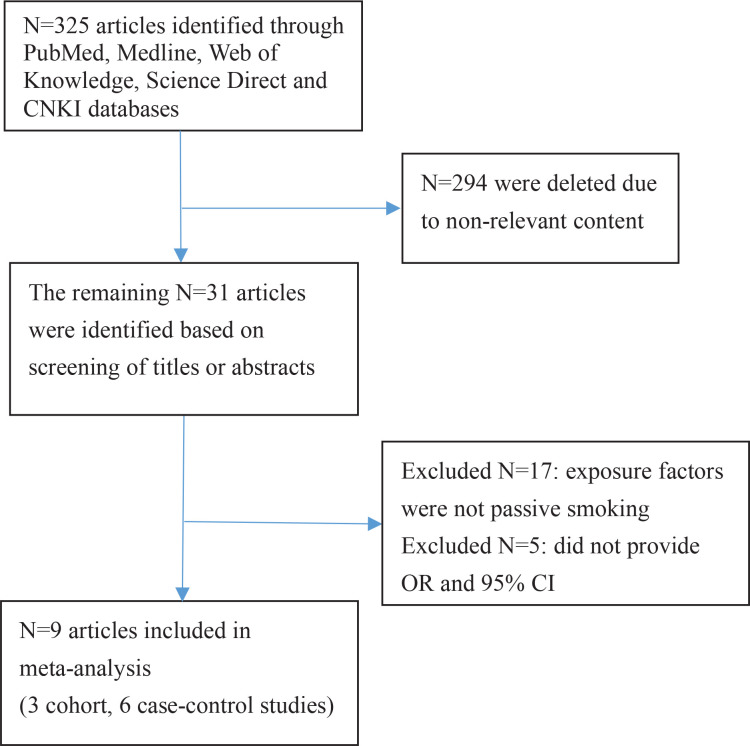
Main characteristics of in cluded studies on the passive smoking and risk of GDM

Figure 1 shows the search process. After reviewing the titles and abstracts of 325 articles, 316 articles that did not meet the inclusion criteria of content, study design, and target population were excluded. A total of nine articles^13-21^ were selected for this meta-analysis; these included 27654 pregnant women, 3730 of whom were diagnosed with GDM; three cohort studies^13-15^, six case-control studies^16-21^; four English articles^13-16^ and five Chinese articles^17-21^; eight study subjects in the Chinese population^13,15-21^, and one in the European population^14^. Seven studies indicated a positive correlation between passive smoking (who were currently exposed to passive smoke but did not actively smoke) and GDM^13-19^, and two did not^20,21^ (Table 1).

**Table 1:** Main characteristics of included studies on the passive smoking and risk of GDM

*Authors Year*	*City Country*	*Survey time*	*Language*	*Study*	*Sample size*	*GDM*	*OR 95 % CI*	*Adjustment factors*	*Score*
Na et al.^13^ 2022	Beijing China	2017–2020	English	Cohort study	3083	562	1.37 (1.11–1.70)	Age, BMI, ethnicity, education level, profession, parity	7
Morales et al.^14^ 2022	Valencia Spain	2/2017–4/2020	English	Cohort study	1262	106	1.66 (1.15–2.38)	Age, BMI	6
Gao et al.^15^ 2020	Tianjin China	10/2010–8/2012	English	Cohort study	19331	1485	1.36 (1.12–1.65)	Age, BMI, family history of diabetes, parity, education level, pressure, number of pregnancies, weight gain during pregnancy, drinking	7
Carroll et al.^16^ 2018	Beijing China	1/2012–6/2014	English	Case-control	276/276	276/274	1.52 (1.05–2.20) 1.71 (1.14–2.56)	Education level, profession, drinking, physical activities, total sleep time, number of pregnancies, family history of diabetes	7
Yang and Zhou^17^ 2018	Linyi China	11/2013–6/2017	Chinese	Case-control	1018	302	1.571 (1.207–1.985)	Age, progestational BMI, number of pregnancies, education level, family history of diabetes, sleeping hours, weight gain during pregnancy, physical activities	7
Shi et al.^18^ 2021	Huzhou China	3/2019–10/2019	Chinese	Case-control	300	200	1.571 (1.199–2.06)	Age, progestational BMI, number of pregnancies, dietary habit, education level, family history of diabetes, sleeping hours, weight gain during pregnancy, physical activities	6
Shu et al.^19^ 2020	Ningbo China	1/2018–3/2019	Chinese	Case-control	1644	672	1.906 (1.501–2.421)	Age, education level, ethnicity, family history of diabetes, pre-pregnancy weight, number of pregnancies, abortion, pressure	7
Ou et al.^20^ 2002	Shanghai China	10/1999–2/2001	Chinese	Case-control	262	85	0.99 (0.352–1.023)	Age, obesity during pregnancy, BMI, parity, family history of diabetes, physical activities, education level, cholesterol, trilaurin	7
Guo and Guo^21^ 2020	Zhengzhou China	1/2020–12/2020	Chinese	Case-control	3343	603	1.135 (0.956–1.349)	Age, BMI, parity, abortion, exfetation, dietary habit, sleeping hours	7

GDM: gestational diabetes mellitus.

### Passive smoking and the risk of GDM

**Figure 2:**
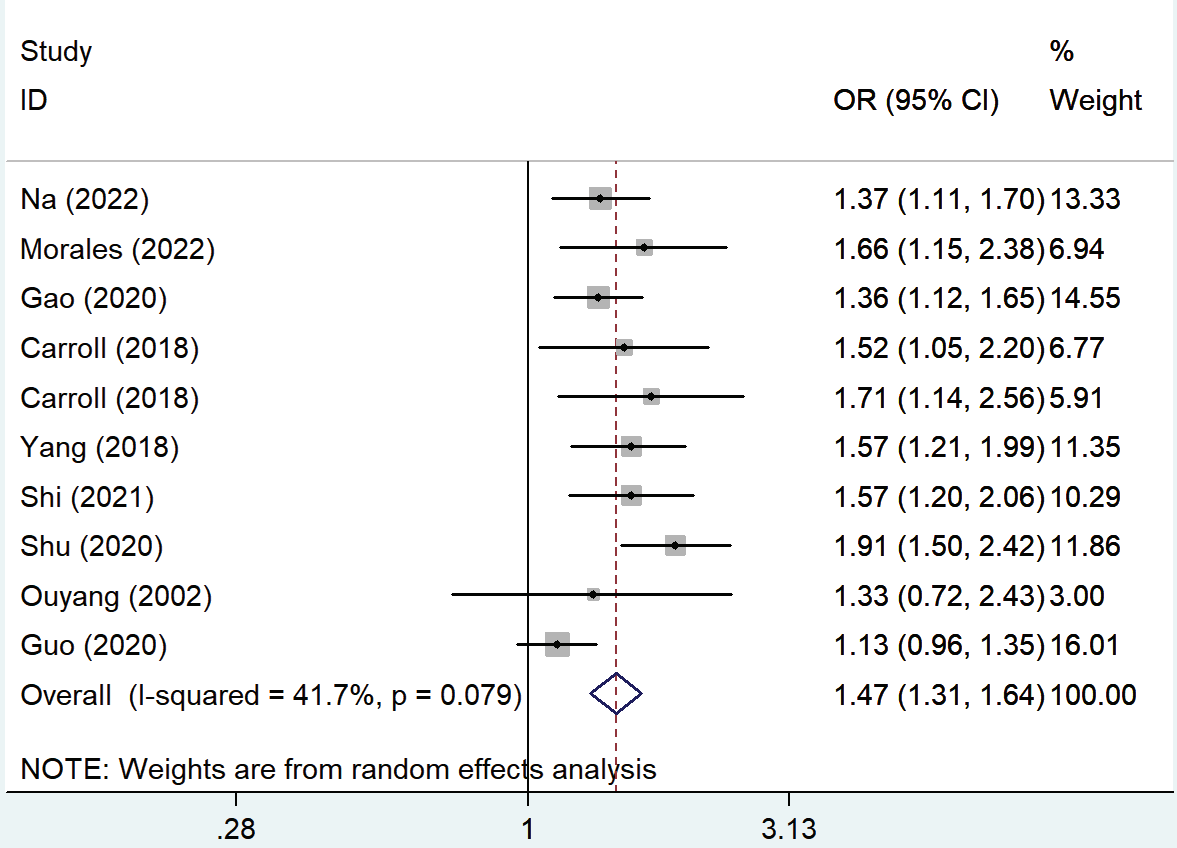
Forest plot of the relationship between passive smoking and GDM

Figure 2 shows the pooled OR values from all studies showing that passive smoking was associated with the risk of developing GDM (OR=1.47; 95% CI: 1.31–1.64) with low heterogeneity (I^2^=41.7%).

### Subgroup and sensitivity analysis

Subgroup analysis based on the study design, language, follow-up years, number of GDM cases, and adjustments to the OR score showed that the results remained similar. Based on study styles, OR values were 1.40 (95% CI: 1.23–1.60, n=3, I^2^=0.0%, p=0.616) for cohort studies and 1.43 (95% CI: 1.30–1.59, n=7, p=0.013) for case-control studies; based upon published in English, 1.44 (95% CI: 1.28–1.62, n=5, I^2^=0.0%, p=0.753), and Chinese, 1.41 (95% CI: 1.26–1.57, n=5, I^2^=73.7%, p=0.004); based upon follow-up years, ≥3 years, 1.48 (95% CI: 1.28–1.72, n=3, I^2^=0.0%, p=0.575) and <3 years, 1.40 (95% CI: 1.27–1.54, n=7, I^2^=61.6%, p=0.016); based upon the number of GDM cases ≥500, 1.36 (95% CI: 1.23–1.50, n=4, I^2^=74.8%, p=0.008) and <500, 1.54 (95% CI: 1.35–1.77, n=6, I^2^=0.0%, p=0.684).

**Figure 3:**
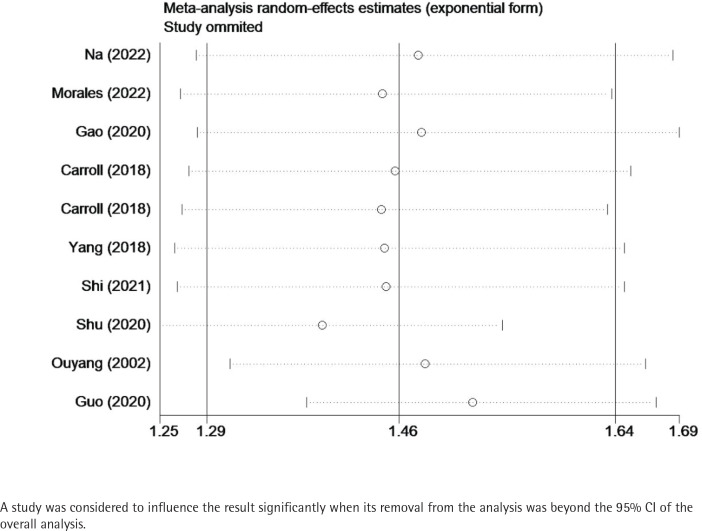
Sensitivity analysis of the relationship between passive smoking and GDM

Sensitivity analysis confirmed that the results remained stable after the removal of one study at a time, in which no individual studies were found to affect the overall OR, and the pooled ORs ranged from 1.44 (95% CI: 1.30–1.60) to 1.55 (95% CI: 1.39–1.72). Table 2 and Figure 3 show the data from our subgroup and sensitivity analyses, respectively.

**Table 2:** Subgroup and sensitivity analysis of the included studies

*Variables*	*Number of studies*	*Effect estimates*		*Heterogeneity*		
		*OR*	*95% CI*	*χ^2^*	*p*	*I^2^ (%)*
**Study design**						
Cohort	3	1.40	1.23–1.60	0.97	0.616	0.00
Case-control	7	1.43	1.30–1.59	16.16	0.013	62.9
**Language**						
English	5	1.44	1.28–1.62	1.94	0.753	0.00
Chinese	5	1.41	1.26–1.57	15.24	0.004	73.7
**Follow-up years**						
≥3	3	1.48	1.28–1.72	1.11	0.575	0.00
<3	7	1.40	1.27–1.54	15.64	0.016	61.6
**Number of GDM**						
≥500	4	1.36	1.23–1.50	11.90	0.008	74.8
<500	6	1.54	1.35–1.77	3.11	0.684	0.00
**Score**						
High	4	1.51	1.35–1.68	5.66	0.129	47.0
Moderate	6	1.33	1.18–1.50	9.30	0.098	46.2

GDM: gestational diabetes mellitus.

### Publication bias

**Figure 4:**
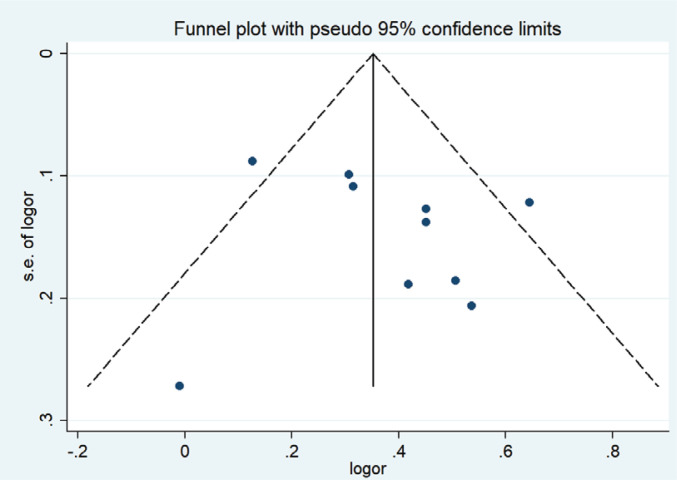
Funnel plot of the relationship between passive smoking and GDM

A funnel plot was used to evaluate publication bias. Begg’s (p= p0.602) and Egger’s (p=0.500) tests showed no publication bias, as shown in Figure 4.

## DISCUSSION

Our meta-analysis confirmed that passive smoking led to a 1.42 times higher risk of pregnant women developing GDM compared to those who had not been exposed to secondhand smoke (OR=1.42; 95% CI: 1.31–1.54, I^2^=47.7%). Because of the heterogeneity, we conducted a subgroup analysis. Sensitivity analysis confirmed that a single study did not alter the pooled OR, and the ORs ranged from 1.44 to 1.55. The global prevalence of GDM is about 1.8–31.0%, and about 20.3% in China^22^. Several epidemiological studies have shown that the etiology of GDM may be a combination of genetic and environmental factors. It is believed that the occurrence of GDM is related to the family history of diabetes, maternal pregnancy age, pre-pregnancy body mass index, and age at first pregnancy^23,24^. Our previous research^8^ confirms that passive smoking is a risk factor for type 2 diabetes mellitus even in those who are not active smokers, but how passive exposure to tobacco smoke leads to GDM is unclear. According to the ‘Chinese reported health hazards of smoking’, the passive smoking rate of fertile women in China was 51.9% in 2012^25^. About 60–75% of non-smoking pregnant women are exposed to smoking environments during pregnancy^26^. A prospective cohort study of 193131 pregnant women in Tianjin^15^ found that 47.3% (9148/19331) of women were exposed to passive smoking during pregnancy, and the risk of GDM caused by passive smoking is 1.36 times higher than that caused by non-passive smoking. Previous studies have confirmed that long-term or passive smoking may affect glucose metabolism and increase the risk of developing diabetes in the population. The pathogenic mechanism is still unclear, but the reason may be that nicotine in tobacco can cause impaired insulin sensitivity and pancreatic islet β-cell function^27^. This results in a sympathetic excitation and increased catecholamine release to antagonize the secretory function of islets^28^. It could also be that the carbon monoxide produced by burning tobacco enters the bloodstream and binds to hemoglobin, leading to an increase in hemoglobin. Epidemiological findings show that women who smoke passively have elevated hemoglobin content and fasting blood glucose levels.

### Limitations

There are some limitations to this study. Only one study reported the exposure of pregnant women to passive smoking in the workplace, and this may have led to an underestimation of the dangers of passive smoking. We did not stratify the analysis by age and weight, but all of the studies are adjusted for age and BMI. The studies used questionnaires to evaluate passive smoking, and self-reported methods could easily result in reporting bias.

## CONCLUSIONS

This meta-analysis indicates that passive smoking increases the risk of developing GDM in non-smoking pregnant women.

## Data Availability

The data supporting this research are available from the authors on reasonable request.
